# Supplementary material for: COVID-19 Vaccination Modifies COVID-19-Related Headache Phenotype: Evidence from Case–Control Study on 309 Participants
Source: Biomedicines. 2025 Nov 27;13(12):2900. doi: 10.3390/biomedicines13122900 (PMC12731189; doi:10.3390/biomedicines13122900)
Supplement: Supplementary file 1 [file biomedicines-13-02900-s001.zip › biomedicines-3955468-supplementary.pdf]

Definition of comorbidities:

- Hypertension (systemic blood pressure higher than 140/90 mmHg in two prior determinations).
- Diabetes (fasting blood glucose >126 mg/dl on two separate tests, HbA1c > 6,5%, blood glucose level >200 mg after oral glucose overload or blood glucose level >200 mg/dl with diabetes symptoms).
- Smoking habit (current or in the preceding 6 months).
- Cardiovascular diseases (coronary artery disease, congenital heart diseases, cardiomyopathies, arrythmias, valvular heart disease, aortic aneurysms and peripheral artery disease).
- Pulmonary diseases (chronic obstructive pulmonary disease (COPD), asthma, occupational lung diseases, interstitial lung diseases and pulmonary hypertension).
- Cancer (any active cancer, excluding epidermoid and basal cell carcinoma).
- Immunocompromised state (congenital or acquired)

Supplementary figure 1:

Duration of the headache, in days, between vaccinated and non-vaccinated patients.

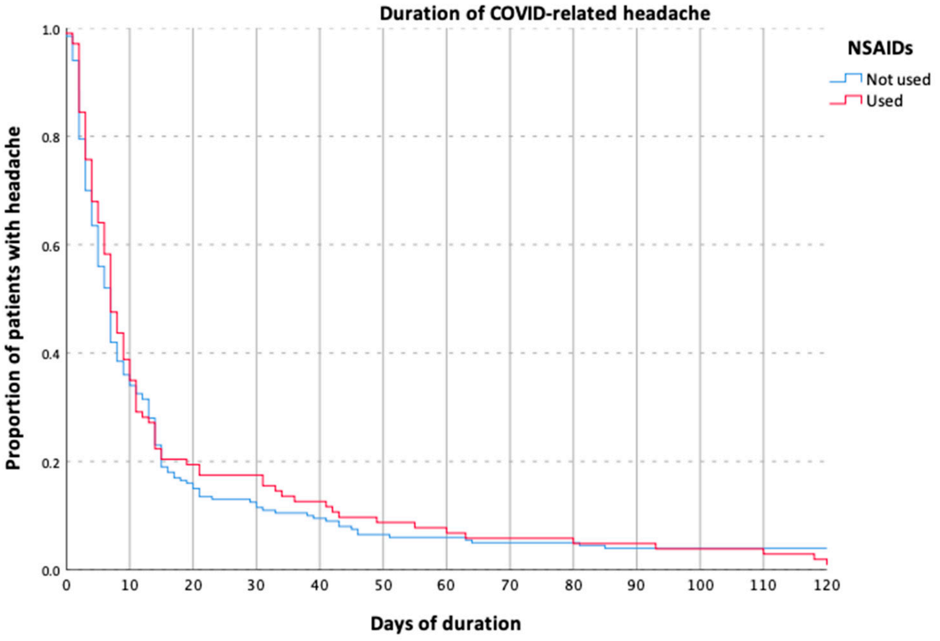

Supplementary table 1: Association between duration of COVID-related headache and prior history of headache:

Variable	Hazard ratio (95% Confidence interval)	P value
Prior history of headache	0.858 (0.663-1.109)	0.242
Prior history of migraine	0.945 (0.657-1.359)	0.761
Prior history of tension-type headache	1.140 (0.762-1.706)	0.523
